# Assessing Advantages and Drawbacks of Rapidly Generated Ultra-Large 3D Breast Cancer Spheroids: Studies with Chemotherapeutics and Nanoparticles

**DOI:** 10.3390/ijms21124413

**Published:** 2020-06-21

**Authors:** Austin R. Holub, Anderson Huo, Kavil Patel, Vishal Thakore, Pranav Chhibber, Folarin Erogbogbo

**Affiliations:** Department of Biomedical Engineering, Charles W. Davidson College of Engineering, San Jose State University, San Jose, CA 95192, USA; austin.holub@sjsu.edu (A.R.H.); andy.huo@sjsu.edu (A.H.); kavil.patel@sjsu.edu (K.P.); vishal.thakore@sjsu.edu (V.T.); pranav.chhibber@sjsu.edu (P.C.)

**Keywords:** spheroids, nanoparticles, doxorubicin, ultra-large spheroids, chemotherapeutics, doxil, breast cancer, triple negative breast cancer, tumor microenvironment

## Abstract

Traditionally, two-dimensional (2D) monolayer cell culture models have been used to study in vitro conditions for their ease of use, simplicity and low cost. However, recently, three-dimensional (3D) cell culture models have been heavily investigated as they provide better physiological relevance for studying various disease behaviors, cellular activity and pharmaceutical interactions. Typically, small-sized tumor spheroid models (100–500 μm) are used to study various biological and physicochemical activities. Larger, millimetric spheroid models are becoming more desirable for simulating native tumor microenvironments (TMEs). Here, we assess the use of ultra-large spheroid models (~2000 μm) generated from scaffolds made from a nozzle-free, ultra-high resolution printer; these models are explored for assessing chemotherapeutic responses with molecular doxorubicin (DOX) and two analogues of Doxil^Ⓡ^ (Dox-NP^Ⓡ^, Doxoves^TM^) on MDA-MB-231 and MCF-7 breast cancer cell lines. To provide a comparative baseline, small spheroid models (~500 μm) were developed using a self-aggregation method of MCF-7 breast cancer cell lines, and underwent similar drug treatments. Analysis of both large and small MCF-7 spheroids revealed that Dox-NP tends to have the highest level of inhibition, followed by molecular doxorubicin and then Doxoves. The experimental advantages and drawbacks of using these types of ultra-large spheroids for cancer research are discussed.

## 1. Introduction

Breast cancer, second only to cancers of the skin, is one of the most prevalent disease types diagnosed in women. According to the latest incident data provided by the Centers for Disease Control and Prevention (CDC), the United States alone reported 124 new cases and 20 deaths per 100,000 women in 2016 [1]. Variance in patient outcome depends on factors such as stage of diagnosis, pre-existing conditions, and cancer subtype; the latter referring to diversity observed in patient-specific tumor cells, where the presence of commonly targeted cell receptors varies [2]. Of most concern is the triple-negative subtype, characteristically lacking genes for expression of estrogen receptor (ER), progesterone receptor (PR), and human epidermal growth factor receptor 2 (HER2) [3,4]. It is thus understood that such variation requires correspondingly specific treatment options per patient. 

Treatment of breast cancer can range from complete breast removal surgery, to less invasive therapies like radiation, hormones, or chemotherapeutics [5,6]. The cost associated with treatment of an individual ranges in correlation with the disease stage, where advanced-stage cancers require more expensive therapies [7]. Because of drug expense and its potential for incompatibility with a given patient, chemotherapy can impart the heaviest physical and financial burdens on both the patient and healthcare system [8]. Therefore, there is a need to more accurately predict chemotherapeutic outcomes and provide patients with personalized treatment options to increase success rates and lower the costs associated with drug development and selection. 

This study is focused on doxorubicin (DOX) as the chemotherapeutic of choice against breast cancer tumor cells. DOX is an anthracycline antibiotic that functions by blocking the topoisomerase-II tumor enzyme responsible for chromosome condensation and segregation, therefore disrupting tumor cell proliferation [9,10]. However, due to the aggressive nature of DOX, patients can suffer from common side effects such as nausea, loss of appetite, hair loss, and physical fatigue [11]. Based on recent advances in nanotechnology, it is understood that such side effects can be reduced via lipid encapsulation of a chemotherapeutic and allow for the introduction of targeted therapies. This encapsulated formulation is commercially available as Doxil^Ⓡ^ [12].

Doxil^Ⓡ^ is a liposomal formulation of DOX commercially available and approved for medical use by the Food and Drug Administration (FDA). Manufactured Doxil^Ⓡ^ has an approximate particle diameter of 85 nm, with narrow size distribution, and a drug encapsulation efficiency of >95%. For this study, commercially available, non-FDA-approved liposomal DOX nanoparticle alternatives Dox-NP^Ⓡ^ and Doxoves^TM^ were employed for chemotherapeutic evaluation. Dox-NP^Ⓡ^ is a pegylated liposomal formulation of DOX with an approximate particle diameter of 135 nm and a drug-load concentration of 2.2 mg/mL [13]. Doxoves^TM^ is also characteristically similar to Doxil^Ⓡ^, with an approximate particle diameter of 85 nm and drug encapsulation efficiency of >98% [12,14].

Previous work has demonstrated the use of three-dimensional (3D) tumor cell spheroid models to study the efficacy and efficiency of chemotherapeutics in vitro, as they have the potential to represent the architecture and physiological properties characteristic of analogous in vivo tumors [15,16]. When compared to a complementary two-dimensional (2D) monolayer tumor cell culture, it is expected that translation of experimental outcomes to patient-drug interactions can be more confidently estimated due to the improvement of physiological relevance provided through representation of the tumor microenvironment (TME). Typically, spheroid models range in diameter from 100–500 μm and are further referred to as small spheroids throughout this text. In addition, although these small spheroids provide a more relevant TME than 2D cell cultures, their size with respect to in vivo tumors is limiting. Evidence of this limitation is realized by absence or under-representation of vascularization, which has been shown to influence cell proliferation and drug efficacy [17,18], thus, requiring adequate representation for a more accurate in vitro model. 

**Figure 1:**
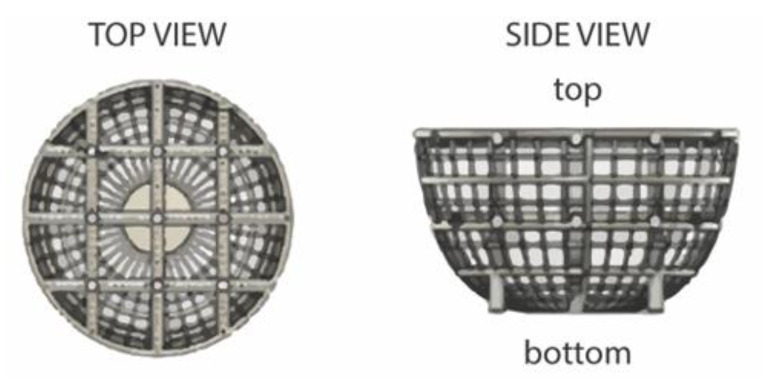
Schematic of ultra-large scaffold showing top view and side view representations [19].

Herein, we investigate a new scaffold technology and the protocol used for generation of ultra-large tumor spheroids (>2000 μm diameter) as a model to study chemotherapeutic and nanoparticle efficacy against breast cancer. This is achieved through assessment of cell culture compatibility, spheroid size, and surface characteristics using image analysis and cell viability. Prellis Biologics “organoid” basket scaffolds, illustrated in Figure 1, were exploited for their ability to promote spheroid development in a reproducible, quantitative manner.

**Figure 2:**
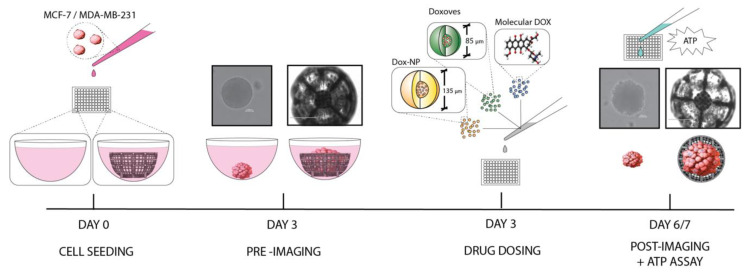
Schematic representation of experimental process.

These oxygen-permeable 3D scaffolds are marketed for their ability to produce sufficient spheroid growth for (i) cell response testing, (ii) animal model transplantation, and (iii) induced cellular differentiation, all within less than 24 h. Furthermore, they are noted to be compatible with 96-well plates and require no special flow systems or media for culture. It is reported that these scaffolds can be used with over 15 primary cell types and a variety of cellular assays for numerous biological research applications [20]. While promising, it is of particular interest to explore this scaffold and culturing technique for use with breast cancer cell lines. Using small spheroids as a comparative baseline, ultra-large breast cancer tumor spheroids were cultured and subjected to chemotherapeutic dosing of DOX, Doxoves^TM^, and Dox-NP^Ⓡ^. Evaluation of spheroid size and cellular activity with respect to treatment versus control allowed for the illustration of the compatibility and limitations of the ultra-large scaffold model. This process is illustrated in Figure 2.

## 2. Results and Discussion

### 2.1. Preliminary Cell Line Evaluation

**Figure 3:**
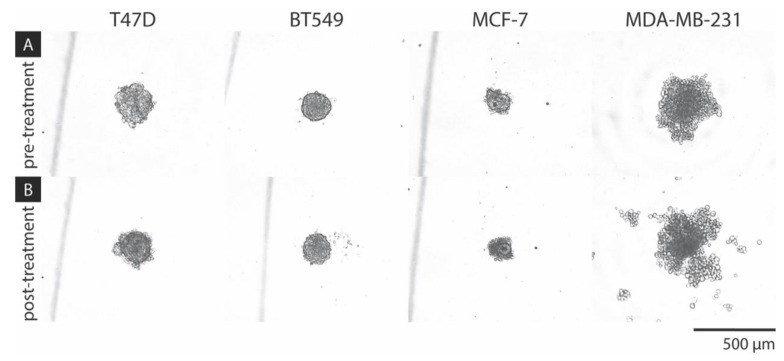
Breast cancer cell line aggregate test spheroids (**A**) 24 h post-cell seeding and (**B**) 96 h post-DOX treatment (5 μM), taken at 5x objective.

A series of separate preliminary experiments were conducted to examine the feasibility of generating reproducible spheroids using a variety of breast cancer cell-lines, including T47D, BT549, MCF-7, and MDA-MB-231. It was found that MCF-7 produced small spheroids with the most consistency in size and observable morphology. Meanwhile, MDA-MB-231 was observed to erratically proliferate overtime, as illustrated in Figure 3 [21,22]. This absence of structure is thought to be the result of a metastatic characteristic of the MDA-MB-231 cell line. The difference in spheroid formation observed on the small scale may have implications for generation of spheroids at a larger scale and was further considered in the design of this experiment. It is recognized that the differences in spheroid formation on the small scale may have implications for creating spheroids on the larger scale; therefore, MCF-7 was chosen as the model cell line for this experiment in order to maintain a quantifiable data set.

### 2.2. Chemotherapeutic Effects on Small Spheroid Cell Proliferation

**Figure 4:**
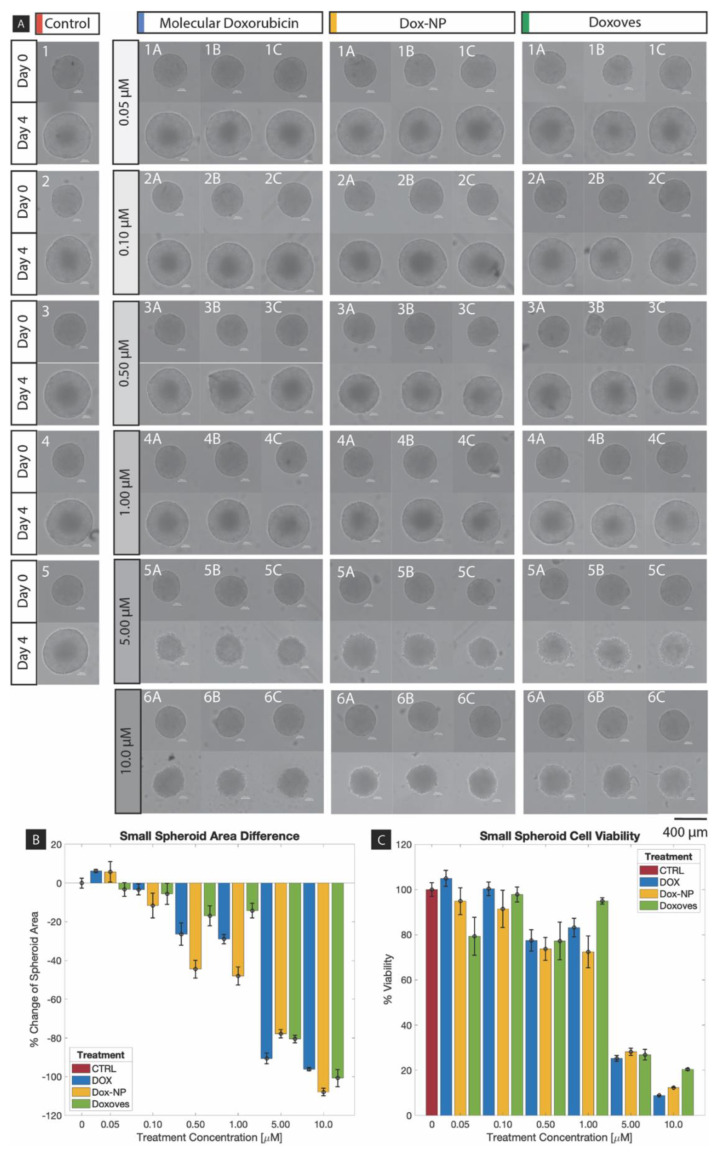
Small spheroid (**A**) Brightfield images of chemotherapeutic response, (**B**) percent change in area, and (**C**) percent viability.

Small MCF-7 spheroids were subjected to chemotherapeutic treatment as a comparative basis for evaluation of the ultra-large spheroid model. Figure 4A displays an experimental set of the small spheroids dosed with the varied concentrations of each chemotherapeutic compared to a control set.

Here, Day 0 corresponds to assembled spheroids preceding drug dosing, while Day 4 corresponds to spheroid outcome approximately 96 h following. Spheroids at Day 0 exhibit a smooth, compact morphology representative of the desired TME formation. The growth seen in the control group at Day 4 demonstrates unperturbed MCF-7 proliferation and is noted for sustaining a more-complete periphery. In the dosed spheroids at Day 4, it is clear that each of the three chemotherapeutics affected cell proliferation and peripheral cell interactions at higher concentrations (5, 10 μM), while lower concentration doses produced less-apparent growth inhibition with respect to the control group. At the median concentrations 0.5 and 1 µM, Figure 4A suggests Dox-NP^Ⓡ^ was most effective at inhibiting spheroid expansion. At 5 µM, molecular DOX appeared to reduce spheroid diameter most significantly compared to the nanoparticle variants. It is hypothesized that passive nanoparticle drug release may have contributed to this result, as the molecular free drug is more readily available for interaction with the tumor cells. 

To assess proliferation quantitatively, spheroid area was measured using ImageJ analysis. The change in area over the four-day culture was calculated from these measurements and are displayed in Figure 4B. This graph represents this change as a percent of average change observed in the control group, per concentration per drug, with error bars representing standard error. As so, average growth of the control group (CTRL) is normalized to 0%. For all three drugs, the result shows a trend of reduced area with respect to increased concentration. More so, treatment with Dox-NP^Ⓡ^ produced the greatest average reduction of spheroid area at four of the six concentrations. 

Supplementary quantitative assessment of cell proliferation was provided using luminescence measurements following adenosine triphosphate (ATP) viability assay application. Resulting viability was again measured as a percent of the control average, where control viability is normalized to 100%, and is displayed in Figure 4C. Here, the error bars again represent standard error of each test group. The trend observed in the dosed spheroids compliments that of the spheroid area processing, where viability predominantly decreases with respect to increased drug concentration. Furthermore, it was noted that nanoparticle variants performed with similar efficacy to the molecular DOX counterpart.

One-way analysis of variance (ANOVA) between the four treatment conditions at each concentration was performed for both MCF-7 spheroid area difference (Figure 4B) and cell viability (Figure 4C) data. For spheroid area, concentrations of 0.50 µM and greater produced statistically significant change (*p* < 0.05). For cell viability, only the concentration of 0.10 µM was not statistically significant. Corresponding *p*-values are listed in Table 1. Complimentary analysis using a Tukey test at a 95% confidence interval was performed for each concentration and can be found in Supplementary Materials section in Figures S1 and S2.

Prior to the result comparison with the ultra-large spheroids, the following observation were the findings within the confine of the small-spheroid experiment:

- When examining change in area over the four-day period, the spheroid size has an inverse relationship with increasing drug concentration for all three drugs. However, a notable observation is that all cancer cell spheroids would increase in size during this period of time with or without the influence (verified by the control) of the drugs. The drugs resulted in retardation to the cellular expansion in proportion to the drug strength; it was theorized that the cell expansion could be attributed by either cellular growth or due to cellular disintegration.
- The viability results of the experiment demonstrated that all three drugs had an inhibitive effect against the cancer spheroids. However, the effects of the drugs did not become significantly apparent until they reached 5 µM in concentration (see Figure 4C).

### 2.3. Chemotherapeutic Effects on Ultra-Large Spheroid Cell Proliferation

**Figure 5:**
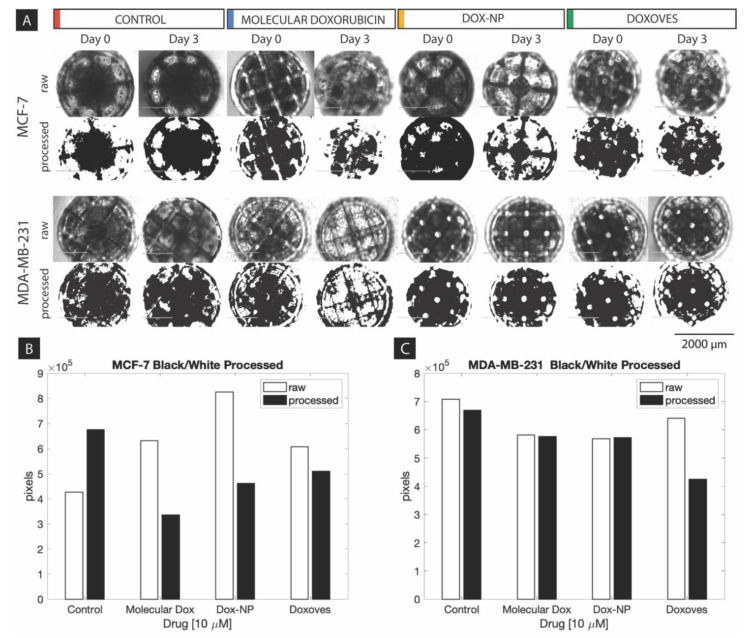
(**A**) EVOS Brightfield microscopic images (“raw”) and binary processed images (“processed”) using ImageJ of ultra-large (2000 μm) MCF-7 and MDA-MB-231 spheroids cultured in Prellis Biologics scaffolds pre- (Day 0) and post- (Day 3) treatment with DOX, Dox-NP^Ⓡ^, and Doxoves^TM^ drug formulations (50 µM). Images taken at 4x objective. (Scale bar 2000 µm). Below are graphical presentations of (**B**) MCF-7 and (**C**) MDA-MB-231 binary processed images shown in (**A**).

Similar to the above study of small spheroids (~500 µm), several experiments were conducted on ultra-large spheroids (~2000 µm) to investigate their viability as a chemotherapeutic testing platform based on spheroid size, ATP activity, and overall morphology. The breast cancer cell lines used in this study were MCF-7 and MDA-MB-231. Scaffolds were generated from a nozzle-free, ultra-high-resolution printer, manufactured by Prellis Biologics, Inc. (Hayward, CA, USA). For this component of the study, 78 ultra-large scaffolds (~2000 µm) were prepared and seeded with each cell line (MCF-7: 36 scaffolds, 3 controls; MDA-MB-231: 36 scaffolds, 3 controls) for culture over an incubation period of three days to study drug concentration effects on ultra-large tumor spheroids. These scaffolds were then treated with the three DOX formulations: molecular DOX, Dox-NP^Ⓡ^, and Doxoves^TM^ (at four concentrations at 0.05 µM, 0.5 µM, 5 µM, and 50 µM). Figure 5A shows results of the raw brightfield images observed for pre- (Day 0) and post- (Day 3) treatment at 50 µM with the aforementioned drug treatment groups versus a control, and their corresponding binary processed images (via ImageJ) for quantification. These raw images were captured immediately prior to treatment of the spheroids with drugs (i.e., Day 0), followed by a 72 h exposure to treatment, and immediately before viability assay (i.e., Day 3) in order to understand drug–spheroid interaction and progression of ultra-large tumor spheroid models.

The “raw” images presented in Figure 5A show a dark contrasting, external, circular border which outlines the top view (see Figure 1) of the ultra-large scaffold framework, and a white outer region representing the concocted DMEM cell media in which the spheroid scaffolds were submerged. Within the scaffold framework, crossbars with white nodes act as auxiliary supports to maintain the “basket-like” appearance of the scaffolds. The dark regions within the scaffold are spheroid formations due to multi-cellular stacking and high cell density; consequently, the darker regions inside the scaffold suggest more cell stacking as compared to lighter regions.

The “processed” images shown in Figure 5A were generated for high-contrast observation and quantitative analysis. The optimal value for each image was chosen as a threshold limit to differentiate between cells and the background (as black and white pixels, respectively). This selection was done using visual analysis of raw images to identify and characterize the cells, artifacts, scaffold, cell media interference, cellular discontinuities in scaffold and microscope light interference. The threshold value was adjusted over a range of 256 bits to reflect the true cell population. These “processed” images show a binary representation of spheroidal activity for greater contrast and quantification of their corresponding raw image based on binary pixel threshold. Once again, there is a white region (cell media) surrounding a dark circular region (scaffold) in which spheroid formation of tumor cells is occurring. Inside this circular region, note the high-contrast dark zones indicative of cell-cell stacking in forming tumor spheroids.

From Figure 5A, the raw image results present a progression from Day 0 (pre-treatment) to Day 3 (post-treatment). Take for example, the raw images for MCF-7 treated with Dox-NP^Ⓡ^. The raw image taken on Day 0 of Dox-NP^Ⓡ^ treatment shows a highly dense MCF-7 cell population indicated by the large dark zones and sparse white zones within the scaffold region. Consequently, the raw image taken on Day 3 of Dox-NP^Ⓡ^ treatment shows a drastic decline of dark zones indicative of minimal MCF-7 cell density as a result of drug treatment on the tumor spheroid. Accordingly, the processed images provide a clearer and quantifiable measure of the aforementioned raw images as graphed in Figure 5B,C.

Consider the binary processed images for the previous raw images example of MCF-7 under Dox-NP^Ⓡ^ treatment. The Day 0 binary processed image shows 67.2% of scaffold coverage within the scaffold; the Day 3 binary processed image is 37.5% of scaffold coverage within the scaffold suggesting a decrease in MCF-7 spheroid due to Dox-NP^Ⓡ^ drug treatment. The percentages were calculated by taking the total number of black pixels (representation of cellular activity) obtained divided by the total pixel count of the scaffold. The complete tabulation of the corresponding binary processed results can be seen in Table 2.

**Figure 6:**
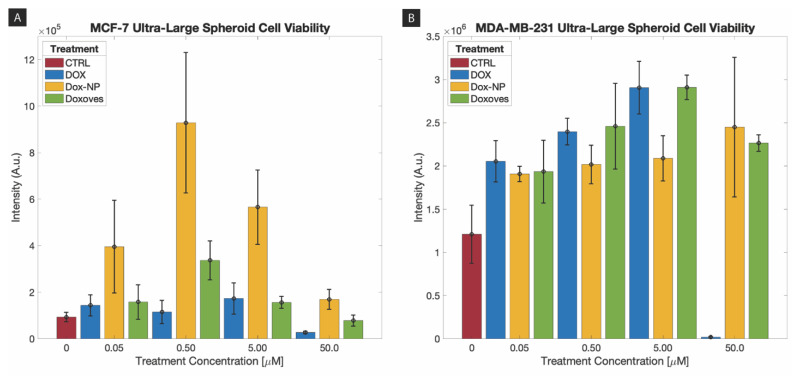
ATP luminescence readings of (**A**) MCF-7 and (**B**) MDA-MB-231 seeded scaffolds exposed molecular DOX, Doxoves^TM^, and Dox-NP^Ⓡ^ drug formulations at concentrations 0.05 µM, 0.5 µM, 5 µM, and 50 µM versus a control.

Following the imaging of samples on Day 3 post-treatment, ATP viability assay was conducted using CellTiter-Glo^Ⓡ^ 3D Cell Viability Assay on 78 ultra-large tumor spheroids (MCF-7: 36 scaffolds, 3 controls; MDA-MB-231: 36 scaffolds, 3 controls). Figure 6 shows the luminescence readings (Intensity, A.U.) for both MCF-7 and MDA-MB-231 triplicate samples exposed to drug concentrations of 0.05 µM, 0.5 µM, 5 µM, and 50 µM under four drug treatment groups (i.e., control, molecular DOX, Doxoves^TM^, Dox-NP^Ⓡ^); here, the control group always consisted of no drug treatment. From the culmination of experiments on ultra-large spheroids, it can be observed from Figure 6 that upon post processing, the molecular DOX treatment group demonstrates the highest inhibition (i.e., lowest luminescence) on the MCF-7 cell line, whereas, Dox-NP^Ⓡ^ treatment group shows the least inhibition (i.e., highest luminescence). With regards to the MDA-MB-231 cell line, the results are not as distinct among the treatment groups.

Using Figure 6, luminescence readings can be compared (i) within a single drug concentration across the four treatment groups, (ii) between the two cell line populations for the same drug concentration exposure, and (iii) across all four drug concentrations for a given cell line for the treatment groups. For example, with regards to (i), in Figure 6A, control and treatment groups for 0.05 µM were tested on three replicates. Dox-NP^Ⓡ^ treatment exhibits highest luminescence as compared to control, molecular DOX and Doxoves^TM^ treatment. The luminescence for treated spheroids is generally higher than the control; to note, this should not be the case for an effective assay. Ideally, the control group should represent the highest number of cells, and thus the highest arbitrary luminescence reading for viability. This drawback did not occur with the small spheroids, and therefore, may suggest that this may not be an effective method for rapidly assessing the viability for ultra-large spheroids, but only small spheroids. Now let us consider (ii) with Figure 8A,E (i.e., MCF-7 0.05 µM and MDA-MB-231 0.05 µM). The control groups for both cell types have similar readings, as do the molecular DOX and DoxovesTM treatment groups; Dox-NPⓇ treatment has consistently higher luminescence for MCF-7 cell types, whereas it is difficult to claim if any particular treatment group is superior in the MDA-MB-231 cell type. Finally, let us take Figure 8A–D as an example for (iii), luminescence readings for MCF-7 ultra-large spheroids under the three treatment groups and a control group at the four drug concentrations (0.05 µM, 0.5 µM, 5 µM, and 50 µM). We can observe here that all triplicates of the MCF-7 sample follow a similar trend of luminescence readings compared to any other treatment group. Overall, triplicates for Dox-NP^Ⓡ^ for MCF-7 show the highest luminescence readings consistently throughout all concentrations for MCF-7 samples. Therefore, according to Figure 6, a standard interpretation of the luminescence readings would indicate that Dox-NP^Ⓡ^ has the least inhibitive activity out of the treatments used for MCF-7 spheroids; MCF-7 cells tend to form uniform and regular spheroids, and due to these characteristics, it may contribute to the consistency in drug (and ATP reagent) results. Conversely, for MDA-MB-231 samples, all three drug treatment groups show minimal differences in effects, suggesting that due to the irregularity in MDA-MB-231 spheroid formation behavior, drug (and ATP reagent) results consistency poses difficulty.

One-way ANOVA between the four treatment conditions at each concentration was performed for both MCF-7 and MDA-MB-231 spheroid cell viability (Figure 6) data. For spheroid cell viability, concentrations of 0.50 µM, 5.00 µM and 50.0 µM for both cell lines were statistically significant (*p* < 0.05). Corresponding *p*-values are listed in Table 3 per cell line samples. Complimentary analysis using a Tukey test at a 95% confidence interval was performed for each concentration and cell line sample which can be found in the Supplementary Materials section in Figure S3.

Figure 5A and Table 2 show cell viability approximations based on ImageJ thresholding analysis and serve as an observation of the treatment effectivity of the drug formulations on both the MCF-7 and MDA-MB-231. However, the graphical results obtained from ATP quantitative viability assessments in Figure 6 present an unsatisfying narrative that does not completely align with the image-based viability results found in Figure 5A. For example, from Figure 5A and Table 2, Dox-NP^Ⓡ^ demonstrates a decrease in MCF-7 spheroid size from 67.2% (Day 0) to 37.5% (Day 3) due to drug treatment; however, from Figure 6, Dox-NP^Ⓡ^ shows highest luminescence readings (i.e., high ATP activity) on MCF-7 spheroids among all treatment groups. Major factors that contribute to the shortcoming of the ATP viability assays may be based on (1) inherent luminescent properties of drugs and nanoparticles that reflect luminescence other than that of cell viability, and (2) impenetrability of ATP reagent to effectively diffuse through highly dense cell spheroids.

To address factors concerning (1) the luminescent properties of the drugs and nanoparticles and (2) impenetrability of ATP reagent as mentioned in the previous paragraph, a short series of experiments were conducted to test the penetration of ATP reagent in 2D and 3D cell culture before testing the drug effectivity on ultra-large spheroids. Known and similar cell concentrations were seeded in a well and on a scaffold. ATP reagent was added to both wells containing 2D and 3D culture. The ATP reading obtained for the 2D well was higher than that of the 3D well. Despite similar cell seeding concentrations, the ATP results varied significantly for 3D culture compared to 2D culture. This information substantiated and guided our rationale for mentioning the factor (2) (i.e., impenetrability of ATP reagent) in the previous paragraph and relying more on the imaging results in Figure 5A for a reliable conclusion. Additionally, to address factor (1) (i.e., luminescent properties of drugs and nanoparticles) scaffolds were pre-washed using PBS buffer prior to ATP assay in order to minimize the effects of drug on luminescence readings.

### 2.4. Cellular Hoechst 33,342 Fluorescence Interaction with Ultra-Large Basket Scaffold

**Figure 7:**
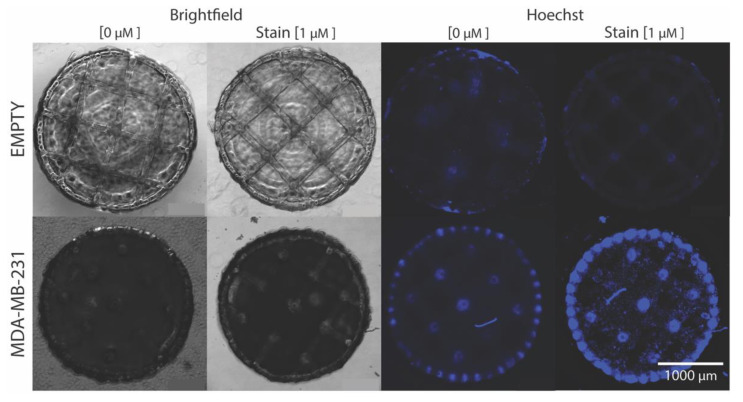
Live MDA-MB-231 seeded basket imaged with a Zeiss AXIO Observer 5X objective. Cellular fluorescence signal (blue) from Hoechst stain highlights cellular integration to the basket structure.

Hoechst 33,342 stain is a widely used fluorescence cell nuclei tagging tool for live and fixed cell culture. To demonstrate cell-to-scaffold attachment and fluorescence signal collection, Hoechst stain was applied to a live MDA-MB-231 seeded basket shown in Figure 7. Additionally, baseline images are included to establish differences to pre- and post-Hosechst stain application. The brightfield (BF) images of the empty basket shows no difference between the images. However, in the blue channel, some auto-fluorescence from the basket structure is present in the pre-stain image, which is then noticeably quenched in the post-stain image. The live BF MDA-MB-231 seeded basket images show little difference between pre-stain and post-stain. There is a distinct contrast difference between the BF cell seeded baskets and the pre-seed baskets. The high density attached cells block transmitted light, eliciting a darker less, discernible basket structure. Here, pockets lacking cell attachment or with a lower cell density can be seen as slightly brighter areas on the basket due to better light penetration. The blue channel non-stained image highlights more of the basket structure due to magnified cell autofluorescence. Cells seem to densely bind to the basket periphery and surface nodes, while the basket material properties also seem to act as a lens concentrating the weak auto-fluorescent signal. Post-stain images reveal stained cells bound to the surface and peripheral scaffold structures. Areas of larger light signal (gray regions) in the BF image correlates to gaps in the cell fluorescence demonstrating pockets of little-to-no cell attachment. 

### 2.5. Spheroid Formation Analysis 

**Figure 8:**
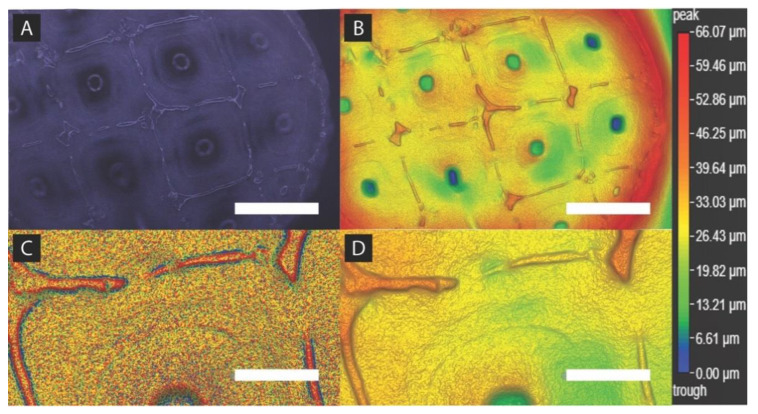
Computational analysis of MDA-MB-231 seeded scaffold surface. (**A**) Seeded basket true color dark field image at 5× objective (scale bar: 500 µm), (**B**) seeded basket colorized image for holistic/global height variation at 5× objective (scale bar: 500 µm), (**C**) seeded basket colorized image for localized height variation at 85% digital zoom and 5× objective (scale bar: 150 µm), (**D**) seeded basket colorized image for holistic height variation at 85% digital zoom and 5× objective (scale bar: 150 µm).

Figure 8 is a demonstration of MDA-MB-231 basket attachment and tumor spheroid formation using BrighTex Bio-Photonic’s ClarityPro dark-field 3D imaging technique (San Jose, CA, USA). With the images taken in Figure 8, spheroid formation and cellular infill within the scaffold structure was demonstrated. Figure 8A shows the true color image captured of a paraformaldehyde (PFA) fixed MDA-MB-231 seeded spheroid surface. The scaffold geometric pattern and periphery is notably discernible while also showing cellular infill. To better visualize cell infill, a colorized global height variation of the same Figure 8A image is shown in Figure 8B. Height variation between the scaffold square geometric structure and the gaps between those structures demonstrates a heterogeneous cell layer formation that dips towards the center. Localized height variation in Figure 8C shows a digitally zoomed textured epithelial layer formation non-uniformly stemming off from the basket structure. Here, MDA-MB-231 cells are forming a highly textured surface between and around the scaffold structure. Global height variation, digitally zoomed in Figure 8D, displays a non-uniform layered cell attachment height that sinks several microns as the cell’s position further away from the basket’s main structure. A sudden dip of about 10–30 µm is located in the center of the basket’s interior square geometry about 200 µm from the closest structure. 

## 3. Materials and Methods

### 3.1. Culture Preparation and Cell Spheroid Placement 

Human Caucasian breast adenocarcinoma breast cancer cell lines Michigan Cancer Foundation-7 (MCF-7) (ATCC^®^ HTB-22™, Manassas, VA, USA) and MD. Anderson-Metastatic Breast-231 (MDA-MB-231) (ATCC^®^ HTB-26™, Manassas, VA, USA) were used in this study for the formation of both small and ultra-large, homogeneous tumor spheroids. Small spheroids were prepared through self-assembly under the following conditions. MCF-7 cells were seeded at 1000 cells per well in an ultra-low attachment 96-well plate, each containing 100 μL of Dulbecco’s Modified Eagle Medium 1X (DMEM) (Gibco ThermoFisher Scientific, Waltham, MA, USA) with 10% Fetal Bovine Serum (FBS) (ATCC^®^, Manassas, VA, USA). Cultures were then incubated at 37 °C for 72 h prior to drug application. At the end of the 72 h incubation period, a pre-treatment imaging on the 96-well plate took place to capture the cell conditions.

With respect to the ultra-large spheroids, prior to scaffold usage, MCF-7 and MDA-MB-231 cell lines were prepared using standard cell culture protocols, and maintained in cell culture media (DMEM, 10% FBS, 1% penicillin-streptomycin with fungicide) in tissue-treated bottom T-75 culture flasks at standard incubation controls (37 °C, 5% CO_2_). The millimetric scaffolds were originally obtained from Prellis Biologics, Inc. (Hayward, CA, USA) and prepared for seeding in 3 mm petri dishes containing DMEM cell culture media; they were later washed with PBS and then coated with poly L-lysine solution to minimize cell spill-out from within the scaffold. Each scaffold was seeded with approximately 2 million cells from the appropriate cell line and incubated at standard cell culture controls (37 °C, 5% CO_2_). After incubation, the petri dishes containing the scaffolds and cells were retrieved and inspected under an EVOS upright microscope for proper cell growth and spheroid formation over a period of five days.

### 3.2. Drug Preparation and Administration 

Molecular Doxorubicin (DOX) (Medkoo, Morrisville, NC, USA) was evaluated against Doxorubicin nanoparticle variants Dox-NP^Ⓡ^ (Avanti Polar Lipids, Alabaster, AL, USA) and Doxoves^TM^ (FormuMax, Sunnyvale, CA, USA) as chemotherapeutic agents. DOX (manufactured and received as powder form) was initially dissolved and prepared in dimethyl sulfoxide (DMSO), with histidine and sucrose as preservatives; afterwards, the dissolved solution was serially diluted to its desired concentration with cell culture media. Doxoves^TM^ and Dox-NP^Ⓡ^ (manufactured and received as liquid form) were initially diluted with manufacturer suggested buffers (comprised with PBS, histidine, and sucrose) less than 24 h prior to the experiment. This was done in order to ensure the preservation of the drugs during the transportation. They were then further diluted, on Day 0 of the experiment, to their desired concentrations with cell culture media at the time of drug administration. The drugs were prepared less than 24 h prior to culture administration in order to minimize drug instability and degradation. (NOTE: Appropriate precautions were taken with safety masks and PPE, as DOX is known to cause irritation and more severely, cardiotoxicity.)

Similar to the drug preparation for the small spheroids above, DOX (MedKoo), Doxoves^TM^ (FormuMax) and Dox-NP^Ⓡ^ (Avanti) formulations were obtained, prepared, and adjusted to the desired concentrations for administration to the ultra-large spheroid samples; the drugs were prepared 24 h prior to administration. The concentrations used for the ultra-large spheroids were 0.05 μM, 0.5 μM, 5 μM, and 50 μM for three drug formulations. The treated ultra-large spheroid samples were followed over 72 h.

### 3.3. Spheroid Imaging and Drug Treatment

Small spheroid images were captured under a 10x objective lens using the Keyence Fluorescence All-in-One Microscope (Itasca, IL, USA). Pre-treatment images (Day 0) were taken per well, followed directly by application of 100 μL of the pre-determined concentrations of DOX, Dox-NP^Ⓡ^, and Doxoves^TM^ to their pre-assigned position. Cultures were returned to incubation at 37 °C for four days. On Day 4 (>96 h), post-treatment images were then taken under identical settings. 

Ultra-large spheroid images were obtained under a 4x objective lens using an EVOS brightfield microscope. Pre-treatment images (Day 0) were taken per petri dish, followed directly by application of 100 μL of the pre-determined concentrations of DOX, Dox-NP^Ⓡ^, and Doxoves^TM^ to the respective scaffolds as determined by the experimental design. Petri dishes containing scaffold cultures were returned to incubation at 37 °C for three days. On Day 3 (>72 h), post-treatment images were then taken under identical settings.

### 3.4. ATP Viability Assay

Following post-imaging, a proliferation assay was conducted via Promega’s Celltiter-Glo^Ⓡ^ Luminescent Cell Viability Assay (Madison, WI, USA). At 50 µM and 200 µL per well, the end culture 96-well plate was transferred onto a luminesce-compatible well plate and the viability read was captured using the Cytation 5 Cell Imaging Reader (BioTek Instruments, Inc., Winooski, VT, USA). The viability results were then further analyzed using GraphPad Prism 8.3.0 (San Diego, CA, USA).

Similarly, for the ultra-large spheroids, cell viability reagent was prepared according to the CellTiter-Glo^Ⓡ^ 3D Cell Viability Assay protocol [23]. The CellTiter-Glo^Ⓡ^ 3D Reagent was thawed at 4 °C overnight and equilibrated to room temperature prior to use. Samples were transferred from petri dish to a 96-well plate with clear bottom wells and microtissues prior to viability reagent application; the samples were suspended in culture media. After application of CellTiter-Glo^Ⓡ^ 3D Reagent, the plate luminescence was measured by a BioTek Synergy/Neo2 (Winooski, VT, USA) plate reader and recorded.

### 3.5. Size Measurement

ImageJ v1.52u (NIH, Bethesda, MD, USA) open-source software was used to measure the area of the spheroids. Keyence microscope PNG image files were uploaded to ImageJ in their original, identical magnification and file dimensions. The area unit of the ImageJ measurements was recorded and calculated in “pixels” at both Day 0 and Day 4 for comparison of a change in relative size. 

### 3.6. Image Binary Processing

The raw images of seeded ultra-large scaffolds obtained from EVOS Brightfield microscopy were processed using ImageJ v1.52u (NIH, Bethesda, MD, USA) software. The raw true color images were converted to grayscale to identify an optimal threshold value from the 256-bit grayscale histogram plot.

### 3.7. Statistical Analysis

The data for both small and ultra-large spheroid samples were statistically analyzed using Minitab 19 (Penn State Univ., PA, USA) statistical analysis software. The size measurement and cell viability data were normalized into percent difference for all concentrations, and then assessed using Minitab 19 one-way analysis of variance and Tukey with 95% confidence interval (C.I.). 

### 3.8. Hoechst Staining 

Hoechst 33,342 (Life Technologies, Eugene, OR, USA) was diluted in cell culture medium (DMEM, 10% FBS, 1.5% Pen-Strep) to a 1 µM Hoechst concentration. Cell culture medium for the 3D cell cultures were replaced with the Hoechst 33,342 diluted cell culture medium in a 24-well plate and were incubated (37 °C, 5% CO_2_) for 15 min before imaging. 

### 3.9. Dark-Field 3D Imaging

A fixed MDA-MB-231 seeded scaffold was transferred to a flat imaging slide using a modified transfer pipette. The sample was maintained in a PBS droplet during the 5-min image capture process using BrighTex Bio-Photonic’s (BTBP) ClarityPro dark-field 3D imaging technique (San Jose, CA, USA). Images were captured using a 360° illumination source rotation and all images were stitched together using the ClarityPro specialized machine learning algorithm. BTBP’s 3D viewer software was used for image reconstruction, digital zoom, and application of the height variation heat map. Height variation calibration was conducted with a benchmark wafer of known length, width, and height values and was imaged in the same conditions as the scaffold sample.

### 3.10. Overview

For the small spheroid experiments, three consecutive experiments were conducted to ensure repeatability and more accurate analysis. The three experiments consisted of two sets of three drugs at 0.05 µM, 0.5 µM, and 5 µM, along with an untreated cell line as control. The third experiment was conducted with one set of refined range of concentrations at 0.05 µM, 0.1 µM, 0.5 µM, 1.0 µM, and 5.0 µM.

With regard to the ultra-large spheroid study, two cell lines were prepared and seeded onto large millimetric scaffolds for culture and incubation for five days. For each cell line, a design matrix for drug exposure of four treatment groups (i.e., three drugs and one control group) and drug concentrations ranging from 0.05 µM, 0.5 µM, 5 µM, and 50 µM were applied. The effect of the drug treatment groups on the ultra-large spheroids were then comparatively analyzed against the effect of the same drugs on conventional method of self-aggregating small spheroids.

## 4. Conclusions

The drug testing results from image analysis of the large spheroids have a similar trend to those from the small spheroids. The drug formulations have the same general pattern of inhibition. For MCF-7 spheroids, Dox-NP^Ⓡ^ tends to have the highest level of inhibition, followed by molecular doxorubicin and then Doxoves^TM^. While the similarities in trends are promising, there are some advantages and drawbacks to using the ultra-large spheroids that should be highlighted.

The advantages are related to the time it takes to culture the spheroid, the ease with which the cells are cultured and the confinement of cells that would otherwise spread. Many cells can easily be clustered together into a 3D formation within 24 h. Another advantage is that spheroids can be made from cell lines like MDA-MB-231 that do not form spheroids easily. The creation of MDA-MB-231 ultra-large spheroids on scaffolds permitted the full realization of the 3D nature of experiments, where the cell density could be easily observed and captured. This contrasts with the process for creating small spheroids, where the MDA-MB-231 cells spread to create a non-spheroidal shape. Another advantage of using the rapidly generated spheroids is that image analysis can be used to assess therapeutic inhibition to drug exposure. 

Some drawbacks typically associated with new technology are present here. For example, the scaffolds are expensive to make. Broader adoption of the technology may result in a price reduction in the future. Another example of a drawback is that protocols should be optimized for each cell line it is used for. We draw attention to this because the protocols may appear simple, but changes in culture time, cell handling and the extracellular matrix used can yield different results. Lastly, an additional drawback was recognized by light matter interactions. It was found that the larger the spheroid created, the more difficult it becomes for light to penetrate and provide photonic-related information that can be obtained from fluorescence assays.

Although the use of 3D spheroids was found to be advantageous to cancer cell studies, it is recognized that a static environment does not fully represent the factors for cellular growth of these cancer cells. It was observed that the tumor cells grew overtime without the suppression of the drugs; however, it remains unclear how much of the growth was stunted or enhanced as a result of static media supplies. Additionally, the experiments done thus far only considered single cell line cultures for the entire study; co-cultures may also provide more accurate responses to drug exposure. Thus, it is suggested that (1) a dynamic cancer cell environment (i.e., media circulation) and (2) co-culture studies shall further improve the model and provide more insight for future similar studies. This work serves as a foundation for creating ultra-large spheroids that can play a significant role in cancer drug testing.

## Figures and Tables

**Table 1:** ANOVA P-Values for small MCF-7 spheroid area difference and cell viability.

Concentration [µM]	Area Difference	Cell Viability
	*p*-Value	*p*-Value
0.05	0.143	0.005
0.10	0.311	0.694
0.50	0	0.006
1.00	0	0.004
5.00	0	0
10.0	0	0

**Table 2:** Quantification results (%) for spheroid area coverage by cells corresponding to Figure 5A binary processed images of MCF-7 and MDA-MB-231. Results tabulated for pre- (Day 0) and post- (Day 3) treatment with DOX, Dox-NP^Ⓡ^, and Doxoves^TM^ drug formulations.

Cell Type	Data Type	Control		Molecular DOX		Dox-NP^Ⓡ^		Doxoves^TM^	
		Day 0	Day 3	Day 0	Day 3	Day 0	Day 3	Day 0	Day 3
MCF-7	scaffold coverage (% area)	34.7	54.9	51.4	27.3	67.2	37.5	49.4	41.5
	# black pixels	425,922	674,276	631,632	334,961	825,351	460,759	606,451	509,364
MDA-MB-231	scaffold coverage (% area)	57.6	54.4	52.2	34.6	47.2	46.8	46.2	46.5
	# black pixels	707,343	668,893	640,883	424,733	580,588	575,383	568,289	571,259

**Table 3:** ANOVA P-Values for ultra-large MCF-7 and MDA-MB-231 spheroid cell viability.

Concentration [µM]	MCF-7 Cell Viability	MDA-MB-231 Cell Viability
	*p*-Value	*p*-Value
0.05	0.280	0.324
0.50	0.019	0.012
5.00	0.020	0.023
50.0	0.033	0.012

## Supplementary Materials

Supplementary materials can be found at https://www.mdpi.com/1422-0067/21/12/4413/s1.
