# Supplementary material for: Assessing Advantages and Drawbacks of Rapidly Generated Ultra-Large 3D Breast Cancer Spheroids: Studies with Chemotherapeutics and Nanoparticles
Source: Int J Mol Sci. 2020 Jun 21;21(12):4413. doi: 10.3390/ijms21124413 (PMC7352930; doi:10.3390/ijms21124413)
Supplement: Supplementary file 1 [file ijms-21-04413-s001.pdf]

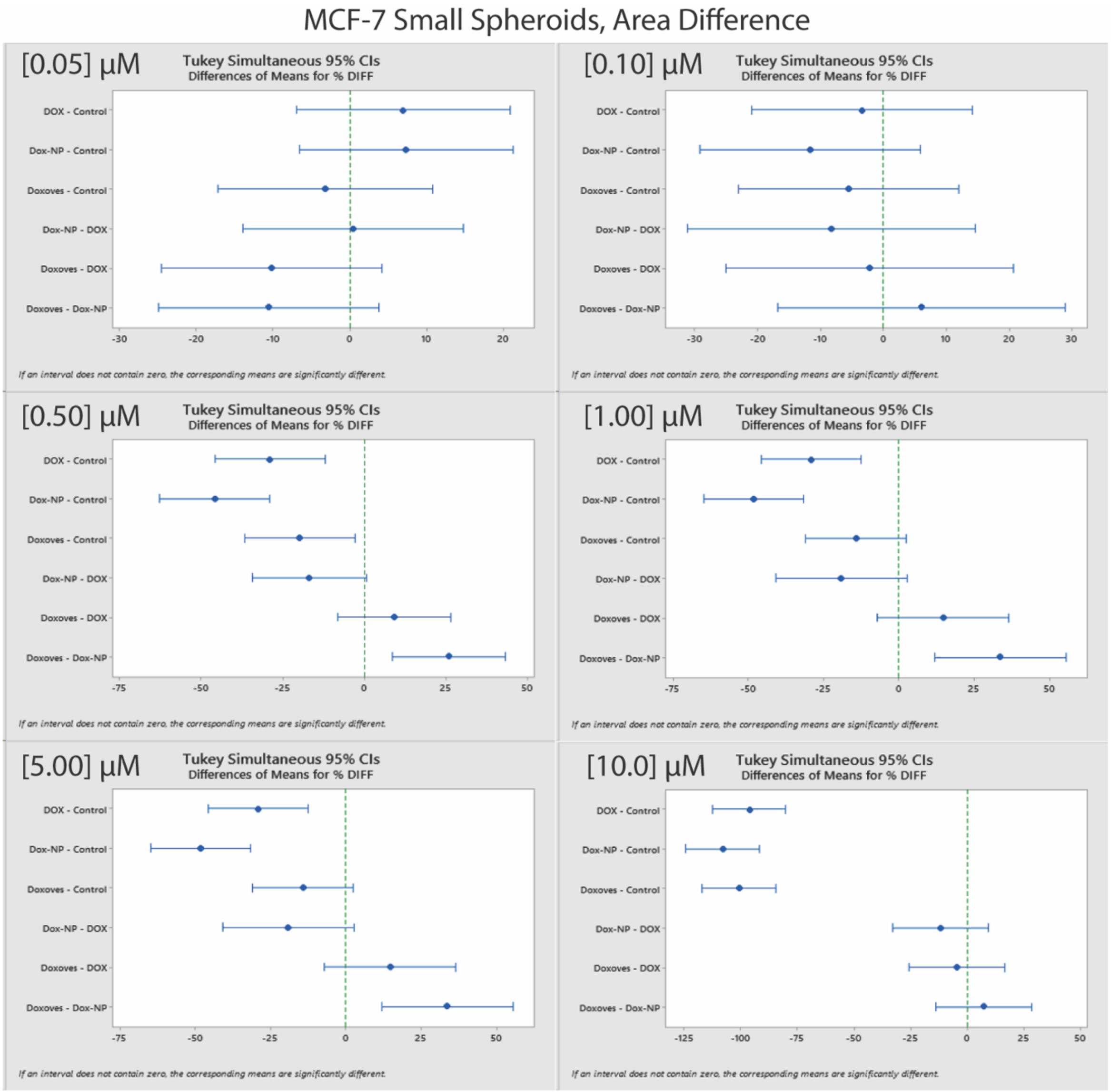

Figure S1. Tukey statistical analysis of change in area of MCF-7 small spheroids at varied treatment concentrations.

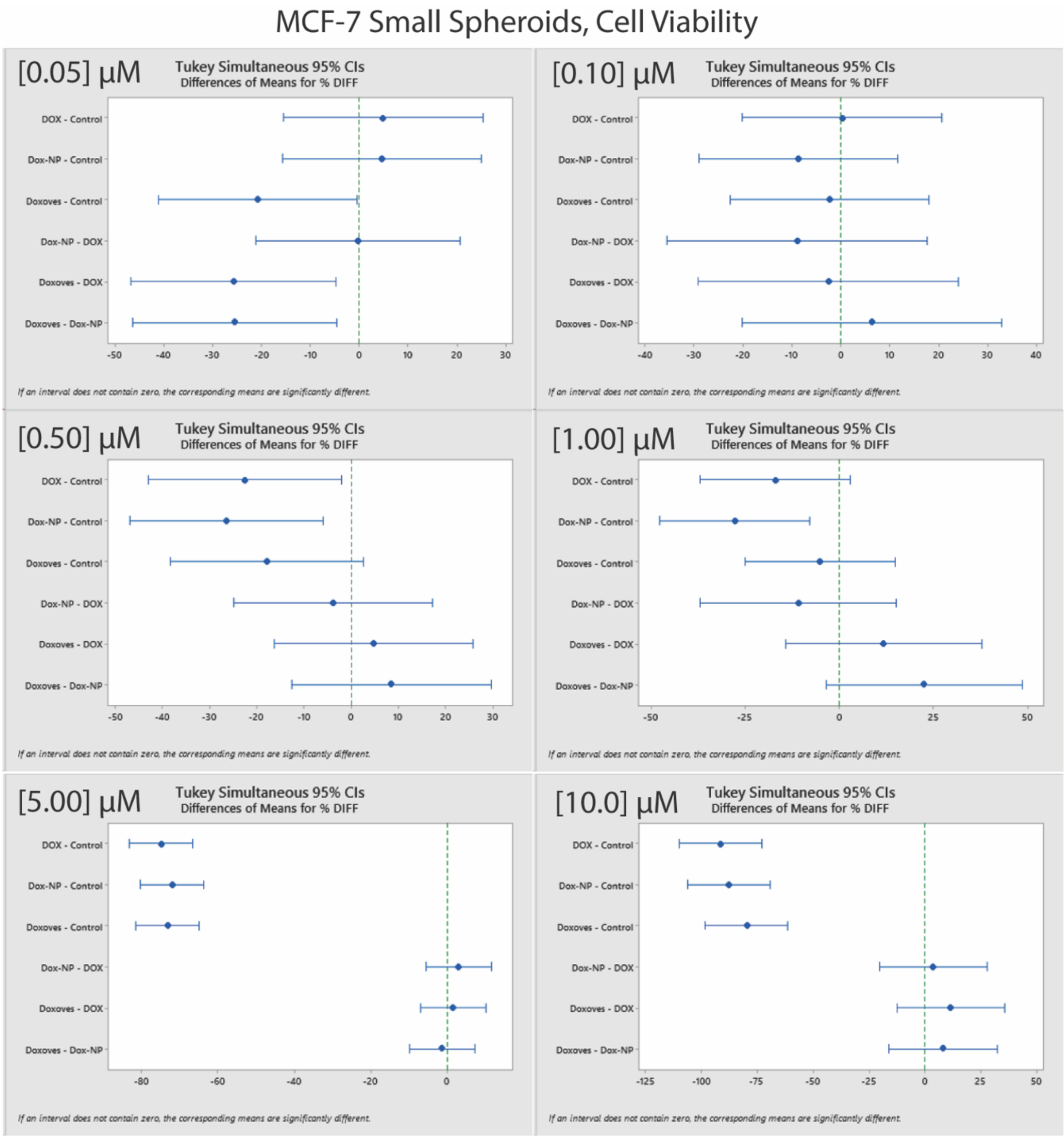

Figure S2. Tukey statistical analysis of cell viability of MCF-7 small spheroids at varied treatment concentrations.

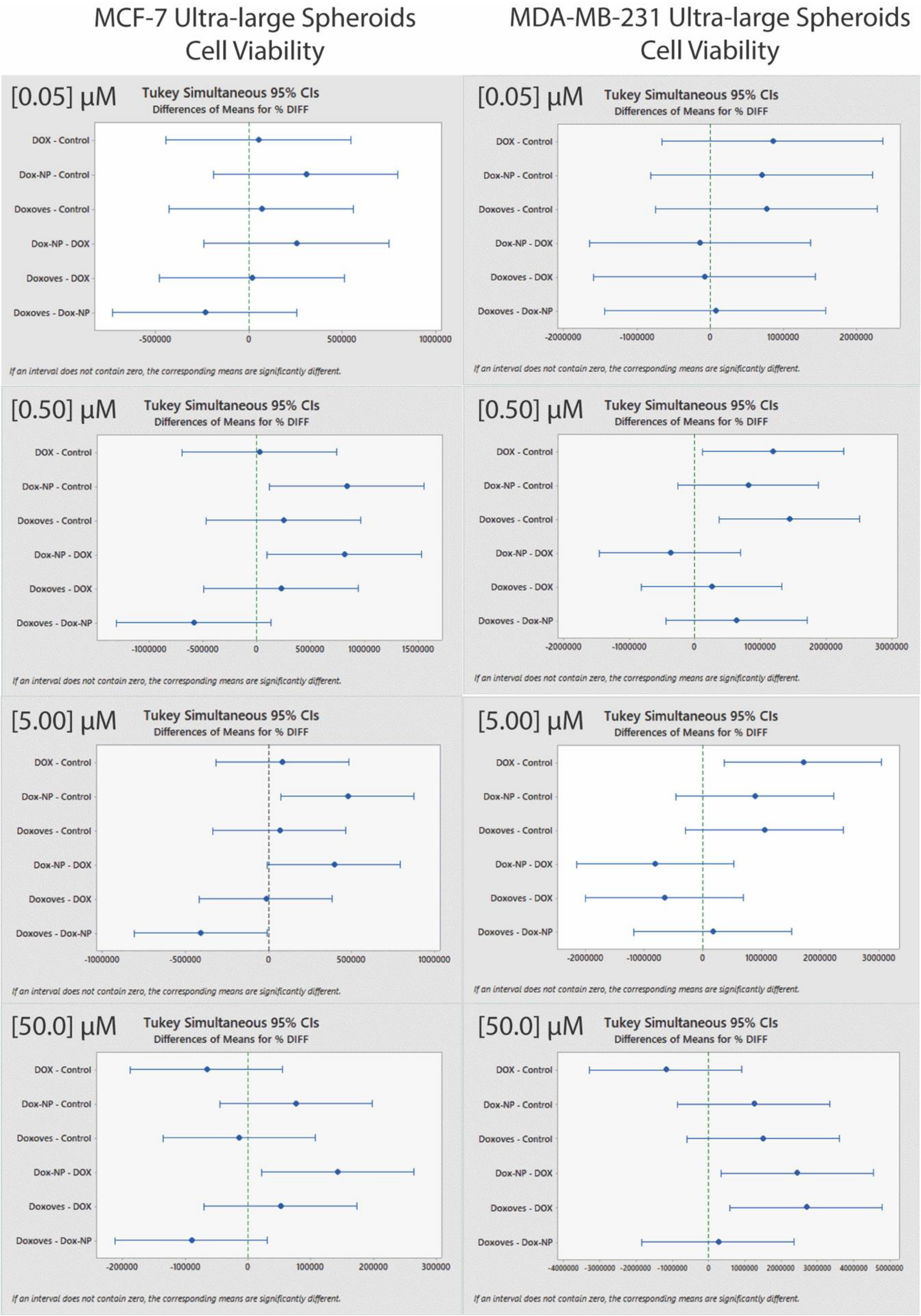

Figure S3. Tukey statistical analysis of cell viability of MCF-7 and MDA-MB-231 ultra-large spheroids.
